# HMGB1–Platelet Interactions: Mechanisms and Targeted Therapy Strategies

**DOI:** 10.1055/a-2622-0074

**Published:** 2025-06-13

**Authors:** Xiyuan Fang, Xianghui Zhou, Xin Zhou, Zhipeng Cheng, Yu Hu

**Affiliations:** 1 Department of Hematology, Union Hospital, Tongji Medical College, Huazhong University of Science and Technology, Wuhan, China, Wuhan, People's Republic of China; 2 Collaborative Innovation Center of Hematology, Huazhong University of Science and Technology, Wuhan, People's Republic of China; 3 Key Laboratory of Biological Targeted Therapy (Huazhong University of Science and Technology), Ministry of Education, Wuhan, Hubei, People's Republic of China; 4 Department of Stomatology, Union Hospital, Tongji Medical College, Huazhong University of Science and Technology, Wuhan, People's Republic of China; 5 School of Stomatology, Tongji Medical College, Huazhong University of Science and Technology, Wuhan, People's Republic of China

**Keywords:** HMGB1, platelet activation, proinflammatory, therapy

## Abstract

Platelets serve not only as crucial hemostatic components but also as pivotal regulators of inflammatory responses, capable of interacting with diverse cell types and secreting abundant extracellular factors. Accumulating evidence demonstrates that high mobility group box 1 (HMGB1), a DNA-binding protein and critical inflammatory mediator, plays multifaceted roles in disease progression, with platelets being one cellular source of circulating HMGB1. Under pathological conditions, platelets release HMGB1 into the extracellular matrix, establishing bidirectional communication between platelets and other immune cells. Moreover, HMGB1 reciprocally activates platelets through Toll-like receptors (TLRs) and receptor for advanced glycation end-products (RAGE), facilitating platelet activation and subsequent release of regulatory factors that drive inflammation-associated pathological thrombosis. In this review, we systematically characterize the HMGB1–platelet axis and elucidate its context-dependent roles in specific disease states. The mechanistic interplay between HMGB1 signaling and platelet pathophysiology is discussed, particularly its implications for disease progression. Furthermore, we critically evaluate therapeutic strategies targeting HMGB1 developed over the past decade, while proposing future directions for dual-target interventions that simultaneously modulate HMGB1 and platelet activity to combat inflammation-driven thrombotic disorders.

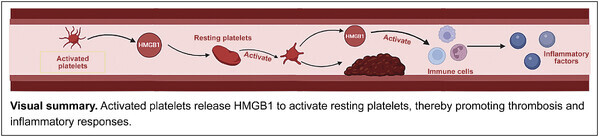

## Introduction

Platelets, as essential hematological components, constitute pivotal mediators in physiological hemostasis. Following vascular endothelial injury, platelets initially adhere to the damaged site through interactions with subendothelial matrix components. The subsequent engagement of surface glycoprotein receptors with collagen or other agonists triggers platelet activation. 1 Activated platelets amplify their recruitment through autocrine and paracrine signaling pathways, ultimately culminating in platelet aggregation 2 and thrombus formation to achieve hemostatic control. Emerging evidence indicates platelets' expanded pathophysiological roles across multiple disease states. Aberrant platelet activation and thrombosis demonstrate prognostic significance in inflammatory disorders, malignancies, and immune-related conditions. 3 Bacterial pathogens can directly interact with platelets to induce activation, thereby facilitating inflammatory progression. 4 In oncological contexts, tumor-associated coagulopathies manifest as hypercoagulable states through mechanisms including: (i) direct procoagulant activity of malignant cells, (ii) suppression of physiological anticoagulant mechanisms, and (iii) pathological thrombosis that critically impacts cancer patient outcomes. 5 Targeted modulation of platelet dysregulation represents a therapeutic imperative for optimizing disease management.

High mobility group box 1 (HMGB1), a 215-amino-acid nuclear protein, 6 also called amphoterin, was initially characterized as a non-histone DNA-binding protein involved in nucleosome stabilization and transcriptional regulation. Structurally, HMGB1 contains two basic DNA-binding domains (HMG boxes A and B) and a highly acidic C-terminal tail that facilitates specific intramolecular interactions. 7 Its biological functions are intrinsically linked to subcellular localization; nuclear HMGB1 maintains chromatin architecture and modulates DNA transcription through coordinated interactions with transcription factors. 8 Cytoplasmic HMGB1 participates in Beclin1-mediated autophagy regulation. 9 Extracellular HMGB1, primarily released through necrosis or secondary apoptosis, 10 11 functions as a canonical damage-associated molecular pattern (DAMP) molecule that orchestrates inflammatory and immune responses. 12 This pleiotropic mediator engages multiple cell surface receptors—particularly Toll-like receptor 2 (TLR2), TLR4, and receptor for advanced glycation end-products (RAGE) 13 —to regulate fundamental biological processes, including autophagy programming, immunogenic cell death (ICD) induction, cytokine/chemokine secretion, inflammatory cell recruitment/adhesion, angiogenesis modulation, and neoplastic cell proliferation/migration. 14 15 16 These mechanisms underpin HMGB1's pathophysiological roles in diverse clinical entities including diabetic complications, septicemia, rheumatoid arthritis, and malignant progression. 17 Emerging research continues to uncover novel HMGB1-mediated pathways, particularly its dual regulatory functions in both physiological homeostasis and disease pathogenesis. The therapeutic potential of HMGB1 modulation warrants systematic exploration through targeted molecular interventions.

In recent years, it has been demonstrated that HMGB1 can also be expressed and released by platelets. It interacts with platelet surface receptors to further amplify platelet activation, establishing its critical role in disease-associated thrombosis. 18 The underlying mechanism involves megakaryocytes synthesizing and transferring both HMGB1 protein and its encoding mRNA to platelets. Upon agonist stimulation, activated platelets upregulate the adhesion molecule CD62P (P-selectin), promoting heterotypic aggregation with neutrophils and monocytes. During this process, HMGB1 translocates from the platelet cytoplasm to the plasma membrane and is subsequently secreted via platelet-derived microparticles (PDMPs) enriched with inflammatory mediators, including HMGB1 itself. These PDMPs interact with receptors on adherent leukocytes and platelets, triggering leukocyte activation and propagating secondary waves of platelet hyperactivity. 19 Targeting the HMGB1–platelet interplay in pathological contexts may yield therapeutic strategies to counteract aberrant platelet activation and platelet-driven inflammatory responses in thrombotic disorders.

**Fig. 1:**
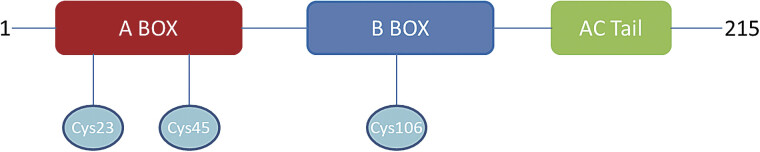
The structure of high mobility group box 1 (HMGB1).

To better understand HMGB1–platelet interactions, this review details HMGB1 signaling pathways in platelets, mainly those related to TLR4 and RAGE, linked to platelet activation and cytokine/chemokine release. As HMGB1 and platelets influence disease progression, we summarize how HMGB1 affects pathological changes in some common diseases through these interactions. However, current antiplatelet therapies do not target HMGB1. So, we review existing anti-HMGB1 treatments. Some can inhibit platelets, and we suggest future research could explore their platelet-related effects. HMGB1 might be a promising new target for antiplatelet therapy ( Fig. 1 ).

### HMGB1 Mediates Platelets Function

#### HMGB1 Modulates Platelet Activation through TLR4-dependent Signaling

Despite their anucleate nature, platelets harbor functionally significant HMGB1 that critically regulates platelet activation and thrombogenesis, making the elucidation of HMGB1-mediated molecular mechanisms governing platelet functionality a paramount research priority.

Early studies revealed that HMGB1 can bind to Toll-like receptors (TLRs), including TLR2 and TLR4, activating downstream molecular pathways (Park). The TLR family, particularly TLR4—a classical pattern recognition receptor (PRR)—serves as a critical mediator of innate immunity and inflammatory regulation. 20 TLR4 is typically activated by pathogen-associated molecular pattern (PAMP) molecules during antibacterial responses, 21 while damage-associated molecular pattern (DAMP) molecules such as HMGB1 and S100 proteins also trigger TLR4 signaling, 22 establishing TLRs as multifunctional regulators in infections, sterile inflammation, and cancer. Current research confirms that platelets express TLR family members, including TLR2, TLR9, and notably TLR4, which is closely associated with normal platelet functionality. 23 The presence of TLR4 receptors indicates that platelets, as essential blood components, extend beyond hemostatic roles to actively participate in inflammatory and immune processes, carrying prognostic significance for disease progression. Consequently, determining whether HMGB1 interacts with platelet-surface TLRs (particularly TLR4) and characterizing their downstream signaling pathways constitute critical research priorities. TLR4 activation by DAMPs induces aggregation of Toll/IL-1 receptor (TIR) domain-containing adaptor proteins, including MyD88, TRIF, and TRAM, 24 initiating downstream phosphorylation events such as NF-κB activation to modulate inflammatory responses. Recent studies demonstrate that during inflammatory or immune challenges, platelet-derived microparticles transport HMGB1 to extracellular spaces while simultaneously displaying it on their surfaces. 25 This platelet-derived HMGB1 exerts autocrine effects by binding to TLR4 receptors on platelets themselves. Subsequent investigations confirmed that HMGB1-TLR4 engagement triggers recruitment of myeloid differentiation primary response gene 88 (MyD88). The intracellular guanylate cyclase (GC)—a key enzyme for cGMP synthesis—then forms a complex with MyD88. Although GC inherently possesses GTP-to-cGMP catalytic capacity, MyD88 is indispensable for this process, as cGMP production initiates only upon complex formation. 26 The generated cGMP acts as a secondary messenger to activate cGMP-dependent protein kinase I (cGKI), which stimulates platelet activation and aggregation, 27 ultimately driving thrombotic events. In conclusion, after being released by activated platelets, HMGB1 can act on other platelets via the TLR4/MyD88/cGKI axis and trigger their activation. This self-reinforcing mechanism promotes widespread platelet aggregation and thrombosis, representing a potential pathway for platelet hyperactivation during inflammatory or immune responses. Targeting this signaling cascade may provide novel therapeutic strategies for preventing pathological thrombosis in inflammatory diseases.

#### The HMGB1/RAGE Axis Regulates Platelet Activation

**Fig. 2:**
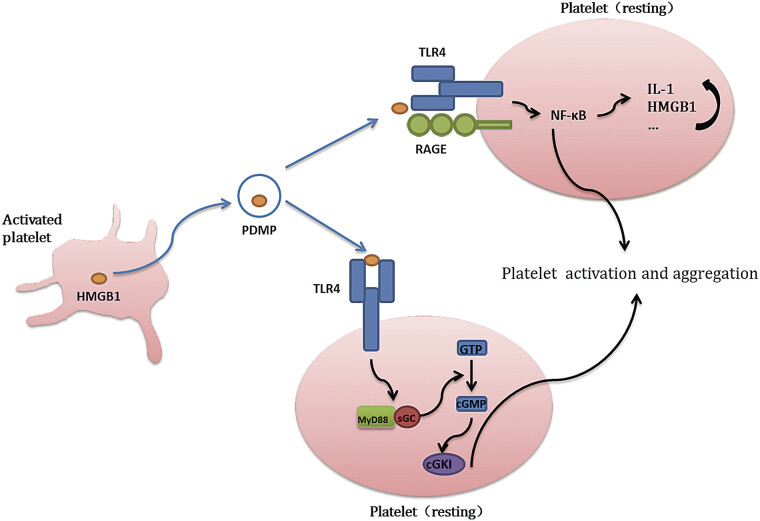
High mobility group box 1 (HMGB1) interacts with platelets through TLR4 and RAGE. PDMP, platelet-derived microparticles.

Advanced glycation end-products (AGEs), glucose–protein complexes typically associated with aging and chronic diseases, interact with their receptor RAGE (receptor for advanced glycation end-products), a transmembrane type I protein belonging to the pattern recognition receptor (PRR) family that mediates innate immune responses. 28 RAGE has been identified on various tissue and cell surfaces. The AGE–RAGE interaction induces proinflammatory cytokine release through mitogen-activated protein (MAP) kinase and nuclear factor κB (NF-κB) pathways. 29 Other ligands such as S100 proteins and HMGB1 have also been shown to bind RAGE, activating NF-κB and modulating inflammatory processes and atherosclerotic plaque formation. 30 31 This suggests that interaction of HMGB1 with platelet-surface RAGE may trigger platelet activation and promote its involvement in inflammatory progression. RAGE expression has been confirmed on platelet membranes, 32 with its levels significantly increasing during platelet activation—primarily through secreted RAGE rather than membrane-bound forms. Membrane RAGE activation further enhances platelet aggregation and functional responses. 33 HMGB1 has been demonstrated to bind platelet-surface RAGE. 34 Upon ligand binding, RAGE undergoes PKCζ-mediated phosphorylation at Ser391 in its cytoplasmic domain, enabling recruitment of adaptor proteins TIRAP and MyD88 to transmit intracellular signals. 35 As previously discussed, MyD88 also serves as an adaptor for TLR4. Subsequent studies reveal that RAGE–MyD88 interaction activates downstream pathways identical to TLR4, including IRAK4 assembly, phosphorylation of Akt and p38, and NF-κB-mediated inflammatory cytokine production. In platelets, HMGB1 simultaneously activates both TLR4 and RAGE, inducing NF-κB pathway activation. This dual activation exerts two critical effects: inflammatory modulation, NF-κB drives platelet release of inflammatory mediators such as IL-1 and upregulates HMGB1/TLR expression, enhancing inflammatory regulation 36 ; and thrombotic promotion, NF-κB activation stimulates platelet hyperreactivity through GPIIb/IIIa activation, facilitating platelet adhesion or aggregation and thrombus formation. 37 38 Additionally, this pathway helps maintain calcium homeostasis, potentially extending platelet lifespan. 39 Although the interplay between RAGE-dependent and TLR4-independent pathways in platelets requires further investigation, the coordinated action of HMGB1 through RAGE and TLR4 undeniably plays a pivotal role in platelet functionality. Elucidating HMGB1–RAGE interactions provides critical insights into platelet behavior during inflammatory diseases, while mapping this signaling axis offers novel therapeutic targets for platelet-related pathologies ( Fig. 2 ).

### HMGB1 Works with Platelets to Regulate Disease Progression

#### Cardiovascular Disease and VTE

##### Cardiovascular Disease

Cardiovascular diseases remain the leading cause of mortality worldwide, with individuals potentially experiencing endogenous or exogenous vascular damage throughout their lifetimes. Major cardiovascular pathologies include atherosclerosis and stroke. 40 Platelets, as essential blood components, serve as pivotal regulators of hemostasis and thrombosis, playing equally critical roles in the progression of these diseases. 41 When vascular endothelial injury occurs and sub-endothelial collagen becomes exposed, platelets interact with collagen through surface glycoproteins (GPs), triggering platelet activation and aggregation that ultimately leads to thrombus formation. 42 The activation of platelets at sites of endothelial damage has been demonstrated to contribute to atherosclerotic progression, with platelets participating in the transition of plaques toward unstable and rupture-prone states. 43 44 This compels us to investigate the interplay between HMGB1 and platelets during atherosclerosis development. Early studies identified vascular smooth muscle cells and monocytes/macrophages as the primary sources of HMGB1 in atherosclerotic lesions, where it predominantly drives inflammatory processes within plaques. 45 Subsequent research confirmed that activated platelets within plaques also release HMGB1, establishing its connection to atherosclerosis via the HMGB1/RAGE pathway. 34 We hypothesize that platelet-derived HMGB1 acts as a proinflammatory factor in atherosclerosis by interacting with RAGE to activate vascular endothelial cells, thereby promoting the release of inflammatory mediators such as ICAM-1, VCAM-1, TNF-α, and IL-8. 14 This interaction also stimulates smooth muscle cell proliferation and migration while modulating monocyte/macrophage functions. 46 Although platelets serve as HMGB1 sources, the potential reciprocal influence of HMGB1–platelet interactions on plaque formation requires further exploration.

In other cardiovascular conditions such as stroke, clinical studies reveal elevated HMGB1 levels in plasma and on platelet surfaces of patients. Animal experiments demonstrate that HMGB1 knockout reduces post-stroke plasma HMGB1 and NETs while improving neurological outcomes, 56 suggesting HMGB1's potential as both a diagnostic biomarker and therapeutic target in thrombotic disorders. Nevertheless, the comprehensive mechanisms by which platelet-derived HMGB1 coordinates inflammatory and thrombotic processes warrant further investigation.

##### VTE

Venous thromboembolism (VTE), encompassing deep vein thrombosis (DVT) and pulmonary embolism (PE), represents a common thrombotic disorder arising from the interplay of innate immunity, sterile inflammation, and thrombosis. 47 48 Initial studies established the regulatory role of platelet-derived HMGB1 in venous thrombosis. Activated platelets release HMGB1, which recruits and activates monocytes via RAGE and TLR2, inhibits monocyte apoptosis, enhances proinflammatory cytokine release, and amplifies platelet aggregation, thereby coordinating sterile inflammation with thrombotic processes. 49 50 Subsequent research refined our understanding of HMGB1's role in venous thrombosis through its mediation of neutrophil extracellular traps (NETs). NETs, initially identified as an antimicrobial mechanism of neutrophils, are formed when activated neutrophils expel nuclear contents—including extracellular DNA, histones, and other nuclear proteins—into the extracellular space. 51 52 53 These structures have since been implicated in thrombotic processes, with platelet-derived HMGB1 shown to regulate NET production. 54 In DVT, platelet-derived HMGB1 recruits neutrophils, potentially through interactions between activated platelet surface CD62P and neutrophils, thereby promoting NET generation. These activated neutrophils and NETs subsequently accelerate thrombus formation—a phenomenon significantly attenuated in HMGB1 knockout mice. 55 Thus, HMGB1 modulates venous thrombosis through dual mechanisms: recruiting monocytes and neutrophils to induce NET-mediated sterile inflammation, while directly promoting platelet aggregation and activation to synchronize inflammatory and thrombotic events.

#### Infectious Disease

In infectious disease research, the focus is often on pathogen identification and early antibiotic therapy. However, in cases involving virulent pathogens, polymicrobial infections, or immune-compromised hosts, the pathogen is no longer the sole concern. Systemic inflammatory dysregulation may progress to multi-organ dysfunction or shock (termed sepsis). 57 Sepsis is characterized by an exaggerated host immune response, frequently accompanied by abnormal coagulation and thrombosis, leading to platelet and clotting factor depletion and ultimately disseminated intravascular coagulation (DIC). Its mechanisms involve three pathways: activation of procoagulant pathways (e.g., monocyte activation), impairment of anticoagulant systems (e.g., thrombomodulin dysfunction), and suppressed fibrinolysis. 58 NETs exhibit dual roles in sepsis—trapping pathogens while simultaneously exacerbating organ damage. 59 In septic conditions, platelets release exosomes carrying diverse molecular cargo including transporting nucleotides, proteins, and signaling molecules that promotes inflammation and organ injury by facilitating intercellular communication. 60 61 Subsequent investigations reveal that such exosomes also contain HMGB1. Platelet-derived HMGB1 within exosomes activates neutrophils by enhancing phosphorylated Akt (p-Akt) and mechanistic target of rapamycin (p-mTOR) expression, thereby amplifying NET release to modulate anti-pathogen inflammatory responses. 62 The multifaceted role of platelet-derived HMGB1 in sepsis extends to monocyte interactions, where it stimulates transcription factor activation and suppresses the thrombomodulin (TM) system, exacerbating microvascular thrombosis. 63 These findings position platelets as inflammatory mediators in infection through exosomal HMGB1-dependent crosstalk with neutrophils and other immune cells. Monitoring platelet-derived HMGB1 levels may provide novel insights into sepsis progression and therapeutic stratification.

This paradigm extends to other infectious diseases. Research shows that high HMGB1 levels in COVID-19 patients are linked to increased platelet activity, indicating it could help monitor clotting risk. 64 Similarly, scrub typhus patients with more severe illness have higher HMGB1, but it is unclear how much comes from platelets. 65 These findings suggest platelet-produced HMGB1 may be useful for evaluating disease severity and platelet function in infections.

#### Autoimmune Disease

Autoimmune diseases, a group of disorders characterized by dysregulated immune activation, aberrant immune cell responses, autoantibody production, excessive inflammatory mediator release, and subsequent self-tissue/organ damage, encompass conditions such as systemic lupus erythematosus (SLE), systemic sclerosis (SSc), and rheumatoid arthritis. Accumulating evidence highlights platelets as pivotal contributors to autoimmune pathogenesis through their role as circulating reservoirs of inflammatory mediators.

In SLE, platelet hyperactivity correlates with disease severity, potentially mediated by circulating anti-phospholipid (aPL) antibodies that disrupt phospholipid-dependent anticoagulant systems and prolong thrombus formation. 66 These antibodies bind activated platelet surface phospholipids, further stimulating platelet activation. Additionally, complement components C2 and C4d deposited on platelets exacerbate platelet hyperactivity and disease progression. 67 Given HMGB1's established role as an inflammatory regulator, investigations propose platelet-derived HMGB1 involvement in SLE pathogenesis. Studies demonstrate elevated circulating HMGB1 levels in SLE patients, positively correlating with TLR-mediated von Willebrand factor (vWF) release. This suggests a self-perpetuating inflammatory cycle where HMGB1 triggers TLR pathways to induce platelet activation, leading to further HMGB1 and inflammatory mediator release. 68 However, the precise mechanisms require validation in larger patient cohorts.

SSc exhibits early platelet activation marked by enhanced aggregation and increased platelet-derived inflammatory mediators. 69 Platelet hyperactivation associates with pulmonary complications in SSc, likely due to the lung's role as a secondary site of platelet biogenesis. Platelet-released inflammatory factors which contain HMGB1 exacerbate pulmonary vascular injury and dysfunction. 70 These HMGB1-carrying microparticles modulate monocyte/lymphocyte adhesion and function, while promoting proinflammatory cytokine secretion, establishing platelets as a primary HMGB1 source linked to SSc disease activity. 18 71

In vitro experiments demonstrate that APS-associated anti-β2-glycoprotein I (anti-β2-GPI) antibodies enhance HMGB1 release from platelets, potentially serving as prognostic biomarkers for thrombotic risk and proinflammatory states. 72 In rheumatoid arthritis and Sjögren's syndrome, HMGB1 correlates with disease progression, though its platelet-mediated mechanisms remain underexplored. 73 74

Autoimmune pathologies feature aberrant platelet activation driven by chronic inflammatory stimuli, with platelets amplifying disease severity through sustained inflammatory mediator release. 75 Current evidence positions HMGB1 as a potential biomarker for monitoring platelet activity in autoimmunity, with therapeutic strategies targeting HMGB1 signaling pathways holding promise for disease modulation.

#### Cancer

Cancer, caused by factors like viral infections and chemical carcinogens, is marked by epigenetic abnormalities. 76 Tumor progression relies on the tumor microenvironment (TME). 77 Studies show RAGE and its ligands are expressed in cancer and TME cells, promoting tumor growth and metastasis via autocrine/paracrine signaling. 78 79 80 81 Concurrently, ligand–platelet interactions induce platelet activation and thrombosis, contributing to the elevated thromboembolic risk in cancer patients. 82

While platelets actively participate in cancer progression, tumor cells reciprocally modulate platelet function through direct activation pathways, establishing cancer-associated hypercoagulable states. Procoagulant molecules within the TME further amplify platelet hyperactivity. 83 84 This bidirectional crosstalk enables platelets to release vesicles that upregulate growth factor expression in tumor cells, while tumor cells exploit platelet activation to facilitate metastasis, vascular adhesion, and endothelial transmigration. 85 Notably, platelets shield tumor cells from immune clearance and mediate vascular endothelial adhesion during metastatic dissemination. 86

To elucidate platelet–TME interactions, proteomic analyses reveal that 77% of tumor-secreted factors exhibit significant enrichment in platelet-derived mediators. 87 Among RAGE ligands within the TME, HMGB1 emerges as a ubiquitously elevated mediator across solid tumors. HMGB1 orchestrates platelet–tumor cell interactions via TLR/RAGE signaling, directly promoting metastatic cascades. 88 Tumor cell-derived HMGB1 further activates platelets through feedback mechanisms, establishing autocrine/paracrine amplification loops. 87 Recent clinical studies demonstrate significantly elevated plasma HMGB1 levels in cancer patients with thrombotic complications compared with those without, correlating with platelet-derived microparticles. 89 These findings position HMGB1 as both a biomarker for thrombotic risk stratification and a therapeutic target to mitigate cancer-associated hypercoagulability.

**Fig. 3:**
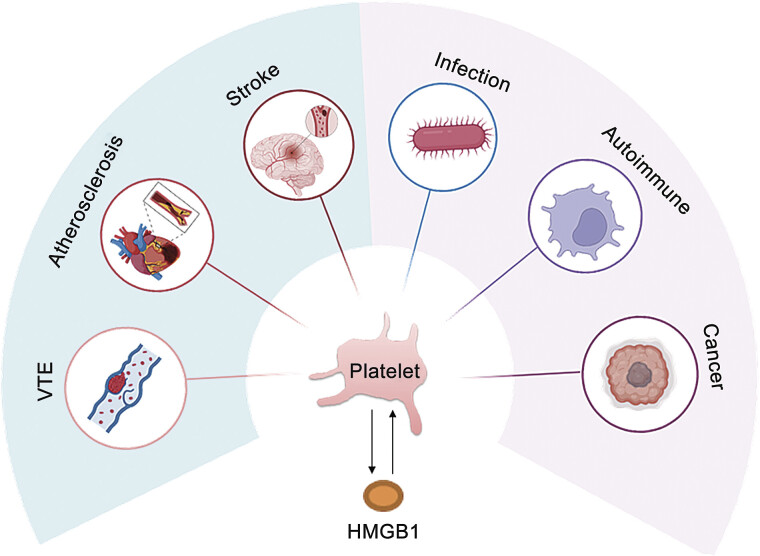
Diseases related to high mobility group box 1 (HMGB1) and platelet. VTE, venous thromboembolism.

In hematologic malignancies, HMGB1–platelet interactions drive excessive inflammatory cytokine release and platelet activation, potentially precipitating disseminated intravascular coagulation. 90 Emerging research implicates HMGB1 in tumor therapy resistance. For instance, anti-CD31 monoclonal antibody therapy fails in neuroblastoma models due to HMGB1-mediated upregulation of epithelial–mesenchymal transition genes under hypoxic TME conditions. 91 As platelets constitute a major HMGB1 source within the TME, targeting HMGB1–platelet crosstalk may overcome therapeutic resistance and improve clinical outcomes ( Fig. 3 ).

### HMGB1 Pathway as a Potential Therapeutic Target for Regulating Platelets

Antithrombotic drugs, used clinically for thrombosis prevention and treatment, fall into two main categories: antiplatelet agents (e.g., aspirin, clopidogrel) work via irreversible COX-1 inhibition to reduce thromboxane production or by blocking ADP receptors, while anticoagulants (e.g., warfarin, novel oral agents) target coagulation factors (e.g., IIa, Xa). Next-generation drugs include potent P2Y12 antagonists (prasugrel), reversible inhibitors like ticagrelor, and direct oral anticoagulants (DOACs), widely applied for thrombotic management across clinical settings. 92

Notwithstanding their widespread clinical deployment, conventional antiplatelet regimens are associated with inherent limitations, including documented incidences of pharmacodynamic resistance—manifesting as attenuated suppression of platelet reactivity—and unintended interference with disease-modifying therapeutic paradigms. 93 These constraints underscore the imperative for innovative therapeutic strategies targeting dysregulated platelet activation pathways.

As previously delineated in our discourse, HMGB1, functioning as a pivotal immunomodulatory entity, engages in bidirectional crosstalk with platelets—a molecular interplay that manifests dual regulatory capacities: thrombotic modulation through platelet activation and inflammatory governance via the induction of proinflammatory mediator synthesis and secretion. Mechanistically, HMGB1–platelet interactions orchestrate a synchronized pathophysiological cascade wherein platelet-derived microparticles synergize with HMGB1-mediated TLR4/RAGE signaling to potentiate both thrombotic inflammation amplification and leukocyte–endothelial adhesion. This reciprocally reinforcing mechanism exhibits pan-pathological relevance, pathognomonically exemplified in atherosclerotic plaque destabilization, sepsis-associated coagulopathy, autoimmune disorder-related microthrombosis, and cancer metastasis-facilitating angiogenic niches. Consequently, HMGB1 emerges as a nexus molecule bridging inflammatory and thrombotic pathways, thereby constituting a therapeutically exploitable target for dual-pathway intervention—simultaneously attenuating inflammatory cascades and suppressing pathological clot propagation through pharmacological disruption of HMGB1–platelet molecular dialogues.

Within the conventional antithrombotic agents, acetylsalicylic acid (ASA, aspirin) retains its foundational status. Beyond its canonical anti-inflammatory, antipyretic, and analgesic properties, 94 aspirin's irreversible acetylation of cyclooxygenase-1 (COX-1) at Ser529 sterically impedes arachidonic acid conversion to prostaglandin H2 (PGH2), thereby abolishing thromboxane A2 (TXA2) synthesis—a potent platelet agonist mediating irreversible platelet aggregation via autocrine amplification loops. This pharmacodynamic profile underpins aspirin's clinical utility in secondary prevention of cerebrovascular accidents and myocardial infarction. 95 Contemporary investigations reveal salicylic acid (SA), aspirin's primary metabolite, functions as a high-affinity HMGB1-binding ligand through stereospecific interactions with box A/B domains. SA–HMGB1 complexation not only neutralizes HMGB1's chemotactic recruitment of myeloid cells via CXCL12 inhibition but also abrogates TLR4/MyD88-dependent NF-κB transactivation. Choi et al achieved this effect by administering 500 mg of aspirin orally to reach an effective plasma SA concentration (IC _50_ 0.45–0.6 mg/L). Notably, this suppression extends to both reduced (Cys23/45 thiol) and oxidized (Cys23-Cys45 disulfide) HMGB1 isoforms, with demonstrated oncostatic efficacy in malignant mesothelioma models through blockade of HMGB1 A BOX. 96 97 Such pharmacological repurposing paradigms underscore the viability of augmenting traditional antiplatelet through HMGB1-targeted molecular engineering, potentially yielding novel hybrid compounds capable of concurrent anti-inflammatory and antithrombotic action.

Over the preceding decade, investigative priorities have predominantly centered on counteracting the proinflammatory ramifications of HMGB1, given its instrumental role as a master cytokine orchestrating inflammatory cascades in sepsis, cerebrovascular pathologies, autoimmune disorders, and oncological contexts. The current therapeutic armamentarium targeting HMGB1 encompasses seven mechanistically distinct stratagems: (i) HMGB1 neutralization: Immunoglobulin-based interventions utilizing monoclonal or polyclonal antibodies achieve extracellular HMGB1 sequestration, thereby attenuating its interaction with neutrophil formyl peptide receptors and monocyte TLR4. 98 99 100 (ii) Competitive receptor antagonism: Recombinant HMGB1 A box polypeptides (rA-box, residues 1–89) function as decoy ligands, competitively inhibiting HMGB1 binding to RAGE/TLR4 through structural mimicry of the native box A domain. Intraperitoneal administration in acute pancreatitis models reduced pancreatic necrosis via suppression of HMGB1-induced IL-1β overexpression. 101 102 (iii) Nuclear retention enforcement: Pharmacological strategies enforcing HMGB1 nuclear localization include ethyl pyruvate, inhibits NF-κB-dependent HMGB1 export via IKKβ suppression, reducing serum HMGB1 in septic models and without influence on steady-state intracellular HMGB1 level 103 ; cisplatin analogs, low-dose carboplatin facilitates HMGB1 nuclear sequestration through DNA platinization, decreasing extracellular HMGB1 in cecal ligation and puncture (CLP) murine models 104 ; gold sodium thiomalate, gold-containing compounds suppress HMGB1 exocytosis in LPS-activated macrophages via thioredoxin reductase inhibition. 105 (iv) Receptor pathway disruption: TLR4 antagonists, TAK-242 covalently modifies Cys747 in TLR4's intracellular domain, abolishing TRIF/TRAM recruitment. Phase II trials demonstrate significant reduction in sepsis-associated thrombocytopenia 24 ; soluble RAGE (sRAGE), engineered variants with enhanced heparin-binding domains (sRAGE-HBD), exhibits 5.3-fold higher HMGB1 affinity, neutralizing most of circulating HMGB1 in atherosclerosis models. 106 Also, it successfully reduced the levels of HMGB1 in hepatocellular carcinoma and inhibited the migration and proliferation of cancer cells 107 ; anti-RAGE antibody, using artificially synthesized HMGB1 monoclonal antibodies or a rat anti-murine RAGE, can effectively improve the survival rate of artificially induced mouse sepsis models. 108 (v) Natural compound utilization: Glycyrrhizin, a triterpenoid saponin from glycyrrhiza glabra, forms high-affinity complexes with both two HMGB1's boxes, inhibiting chemoattractant activity of HMGB1. 109 (vi) Endogenous modulator: Thrombomodulin, whose N-terminal lectin domain binds to HMGB1, reducing the inflammation caused by HMGB1 and RAGE 110 ; neuropeptides, ghrelin (an acylated polypeptide produced by stomach cells), vasoactive intestinal peptide (VIP), and urocortin modulate inflammation and improve sepsis survival of animal model by suppressing HMGB1 secretion. 111 112 (vii) Immunomodulatory approaches: IVIG, high dose intravenous immunoglobulin containing anti-HMGB1 antibodies decreases plasma HMGB1 in a cecal ligation and puncture (CLP)-induced sepsis model 113 ; glucocorticoids, intraarticular injection of triamcinolone hexacetonide reduces inflammation in synovial cell in RA patients 114 ( Table 1 ).

**Table 1:** Potential anti-HMGB1 treatment strategies

Molecule	Mechanism	Clinical potential	Pilot phase
mAb	Neutralizing antibody	Brain inflammatory disease	Biological testing
pAb	Neutralizing antibody	Post-traumatic inflammation	Biological testing
rHMGB1 A box	Antagonist	Acute pancreatitis	Biological testing
Ethyl pyruvate	Release inhibition	Endotoxemia/Sepsis	Biological testing
Cisplatin	Release inhibition	LPS-induced inflammation	Biological testing
Gold salts	Release inhibition	RA	Biological testing
TLR inhibitor	Receptor inhibition	Severe sepsis	Clinical trial
sRAGE	RAGE antagonist	Cancer/Hemorrhagic shock	Biological testing
Glycyrrhizin	Binding and inhibition	Hepatitis	Clinical treatment
Thrombomodulin	Binding and inhibition	Anti-inflammatory	Biological testing
Neuropeptide	Binding and inhibition	Sepsis	Biological testing
IVIG	Immune clearance	Sepsis	Biological testing
Glucocorticosteroid	Immune clearance	RA	Clinical trial

Notes: “Clinical potential” indicates that the treatment has shown efficacy in corresponding animal disease models or cell lines, but it does not imply that it will be the sole therapeutic effect when applied in clinical practice.

“Pilot phase” refers to the experimental research stage investigating the treatment's effect on HMGB1.

Emerging evidence reveals modulatory properties of HMGB1 in conventional chemotherapeutics including triptolid, doxorubicin, berberine, and theophylline; they can modulate inflammation in tumors by inhibiting HMGB1-related pathways and inhibiting HMGB1 release. 115 7-methoxy-3-hydroxy-styrylchromone (C6), a synthetic compound derived from the anticancer drug 3-styrylchromones, demonstrates significant HMGB1 inhibitory effects 116 ; benzyloxycarbonyl-Val-Ala-Asp-fluoromethylketone (Z-VADFMK), a broad-spectrum caspase inhibitor, concentration-dependently suppressed HMGB1 release by reducing NF-κB-dependent translocation of HMGB1 from the nucleus to the cytoplasm in apoptotic cells. 98 The classical drug methotrexate has also been found to inhibit HMGB1 expression. 117 Ginsenoside Rg1 has been verified to suppress HMGB1 along with TLR and RAGE signaling pathways. 118 Certain traditional Chinese medicine formulations, including Huangqi Gegen decoction and San Huang Xiao Yan recipe, have been studied for their regulatory effects on HMGB1-related pathways. 119 120 Parallel investigative endeavors have elucidated heparin's HMGB1-sequestration capacity via electrostatic interactions with box A/B cationic residues, 121 inspiring the development of heparin-functionalized polymethacrylate adsorbents. These extracorporeal devices demonstrate most HMGB1 clearance efficiency within 2 hours, concurrently eliminating platelet factor 4 (PF4) and NETs—a therapeutic modality showing promise in mitigating immunothrombosis during septic shock and COVID-19-associated ARDS. 122 These therapeutic strategies modulate the proinflammatory effects of HMGB1 by targeting either HMGB1 itself or its associated pathways. Considering the synergistic proinflammatory role of platelets and HMGB1 in disease progression, we hypothesize that these treatments may also influence platelet functionality.

As confirmed by recent studies, administration of HMGB1 monoclonal antibodies in murine models effectively ameliorates trauma-induced thrombus formation. 123 Piperlongumine, a pyridone alkaloid isolated from long pepper, inhibits collagen and arachidonic acid agonist induced platelet aggregation, 124 and has also been shown to suppress HMGB1 release, inflammatory effects on human endothelial cells, and HMGB1-mediated leukocyte migration. 125 As evidenced by previous studies, glycyrrhizin mentioned earlier inhibits thrombin-mediated platelet aggregation and prolongs thrombus formation time. 126 We hypothesize that platelets and HMGB1 may function as an integrated entity in pathological contexts, where platelet inhibition implies concurrent HMGB1 suppression. However, existing research rarely addresses the therapeutic target synergy between these two entities. We propose that investigating the efficacy of these anti-HMGB1 therapies on platelets, or exploring effects of existing antiplatelet therapies on HMGB1, could facilitate development of novel pharmaceuticals or provide new targets for anti-inflammatory/antiplatelet treatments, applicable to clinical management of inflammation-associated thrombosis in infections, tumors, and autoimmune diseases. Such bidirectional pharmacodynamic profiling could unveil novel poly-pharmacological agents capable of dual-pathway inhibition. Furthermore, rational drug design leveraging structural homology between HMGB1's box B domain and platelet receptor extracellular motifs may yield bispecific inhibitors with enhanced thrombotic inflammation suppression.

## Future Direction

Previous studies have primarily focused on HMGB1's proinflammatory effects, leading to the development of anti-HMGB1 therapeutic strategies such as mAbs. With the discovery of HMGB1–platelet interactions, some research has begun investigating traditional anticoagulants, including aspirin-class drugs and heparins, on HMGB1 modulation, demonstrating their efficacy in clearing or inhibiting circulating HMGB1 to attenuate its proinflammatory effects. Subsequent investigations could focus on how antiplatelet agents influence platelet-derived HMGB1 levels, as well as how anti-HMGB1 drugs affect platelet functionality and activity. Additionally, considering the impact of HMGB1 isoforms on its activity, as HMGB1 is a composite protein, its isoforms are determined by the redox states of three cysteine residues (C23, C45, C106), where fully oxidized HMGB1 exhibits no significant bioactivity, while distinct redox forms mediate HMGB1 binding to specific dominant receptors. 127 In this context, since HMGB1 primarily interacts with platelet RAGE and TLR4, modulating the redox forms of circulating HMGB1 may allow regulation of its platelet interactions. Regarding HMGB1-targeting receptors TLR4/RAGE, multiple TLR inhibitors are under investigation yet face clinical translation challenges. 24 Considering HMGB1 predominantly acts through platelet TLR4, strategies to precisely target this molecule require further exploration. For RAGE, TTP488, an oral small-molecule inhibitor, has shown laboratory-stage efficacy, 128 but clinical translation hurdles persist, and targeting platelet-specific RAGE demands additional research.

However, treatments targeting HMGB1 are not always beneficial. As mentioned earlier, cisplatin and its analogs can cause kidney damage and ototoxicity during cancer therapy. Studies have shown that these side effects are positively correlated with the HMGB1 pathway, and inhibiting HMGB1-related pathways significantly alleviates these adverse effects. 129 130 Additionally, compounds such as ethyl pyruvate (EP) and glycyrrhizin (GL) may lack specificity in their mechanisms of action. EP exerts its anti-inflammatory effects by inhibiting the NF-κB and inflammasome pathways, so using it as an anti-HMGB1 treatment may lead to unintended outcomes. 103 Although GL can directly bind to and inhibit HMGB1, its affinity is relatively mild, potentially resulting in interactions with other structurally similar proteins. 131

Recent studies indicate HMGB1 does not always synergize with platelets; for instance, in immune thrombocytopenia (ITP), studies have demonstrated an inverse correlation between HMGB1 levels and circulating platelet counts, with natural HMGB1 inhibitor 18β-glycyrrhetinic acid (18β-GA, a glycyrrhizin-derived compound) significantly improving platelet counts in ITP murine models. 132 And a research in non-small cell lung cancer (NSCLC) reveals that resveratrol promotes tumor cell release of HMGB1-enriched extracellular vesicles, thereby increasing reactive oxygen species (ROS) in platelets. Elevated ROS levels enhance platelet ferroptosis while suppressing platelet activity, which is hypothesized to result from prolonged HMGB1 exposure and tumor microenvironment complexity. 133 Thus, the interplay between HMGB1 and platelets remains an underexplored frontier.

These challenges mainly stem from insufficient research on HMGB1's mechanisms of action and its platelet-specific effects. Subsequent studies should prioritize elucidating HMGB1's pathways and its role in platelets, while existing anti-HMGB1 therapies may proceed to clinical trials after further confirmation of their biosafety.

## Conclusion

Platelets not only serve as hemostatic agents in vivo but also act as regulators involved in pathological alterations, linking inflammatory cells and functioning as cytokine sources, with numerous unknown mechanisms still implicated in platelets under pathological conditions. The interplay between HMGB1 and platelets holds significant implications for inflammatory responses and platelet activation under pathological contexts. Focusing on HMGB1–platelet interactions can provide a more comprehensive understanding of inflammatory disease progression and platelet involvement. By inhibiting circulating HMGB1—particularly platelet-interacting and platelet-derived HMGB1—it becomes possible to attenuate inflammatory responses while suppressing HMGB1-mediated platelet activation, demonstrating therapeutic potential for managing inflammation-associated thrombosis. Future studies should prioritize elucidating the mechanistic intricacies of platelet–HMGB1 crosstalk to develop platelet-targeted anti-HMGB1 pharmaceuticals.
